# A Fragile Stronghold: Genomics Reveal Angelshark Population Vulnerability in Corsica, a Key Mediterranean Refuge

**DOI:** 10.1002/ece3.72275

**Published:** 2025-10-13

**Authors:** Nadia Faure, Maurine Vilcot, Franck Pichot, Jean‐Jacques Riutort, Adèle Barroil, Florian Holon, Nicolas Tomasi, David Mouillot, Julie Deter, Stéphanie Manel

**Affiliations:** ^1^ CEFE, Univ Montpellier, CNRS, EPHE‐PSL University, IRD Montpellier France; ^2^ MARBEC, Univ Montpellier, CNRS, IFREMER, IRD Montpellier France; ^3^ Bastia Offshore Fishing Furiani France; ^4^ Andromède Océanologie Mauguio France; ^5^ Parc Naturel Marin du Cap Corse et de L'agriate/Parcu Naturale Marinu di u Capicorsu è di L'agriate, Base Nautique Des Minelli Ville Di Pietrabugno France; ^6^ Institut Universitaire de France Paris France

**Keywords:** bycatch, conservation, effective population size, elasmobranchs, genetic diversity, multiple paternity, site fidelity

## Abstract

Once common in Eastern Atlantic and Mediterranean coastal waters, the angelshark ( *Squatina squatina* ) has disappeared from 90% of its historical geographic range over the last century. Populations have drastically declined, likely due to the combined effects of overfishing, coastal habitat destruction, and the species' slow life history traits. The island of Corsica remains one of the last Mediterranean refuges for this IUCN Critically Endangered species, underscoring the need for conservation action. Given the difficulty of observing this benthic shark, we employed genomic methods to investigate the fine‐scale spatial genetic structure, genetic diversity, and effective population size. Skin samples were opportunistically collected from accidental bycatch of angelsharks by local fishers in eastern Corsica and genotyped for 9699 Single Nucleotide Polymorphisms. We show that these individuals belong to a single population and exhibit high site fidelity, particularly among females, supporting male‐biased dispersal. Genetic relatedness analyses identified 35 close family relationships, with 42% of sampled individuals showing a close relative. Additionally, we revealed multiple paternity within a single litter, suggesting a polyandrous mating system not previously documented in Squatinidae. The estimated effective population size of 290 individuals (95% CI: 209–453) is concerning given the persistent bycatch of hundreds of angelsharks by local artisanal fisheries during the annual spring reproductive aggregation of *Spicara smaris* . Protecting these ephemeral breeding colonies would not only benefit angelsharks but also help sustain numerous other threatened elasmobranchs and commercially important fish species (i.e., *Zeus faber* ). Our findings highlight the value of integrating genomic tools into the conservation of elusive marine species. Conservation efforts should focus on reducing bycatch through gear modifications, seasonal fishing restrictions, and preserving estuaries. Studying and protecting this Corsican refuge is of paramount importance, as it could serve as a source population for restoring angelshark populations in formerly abundant areas.

## Graphical abstract

This first genomic insight into a key Mediterranean angelshark stronghold reveals an effective population size below critical conservation thresholds, high site fidelity, and multiple paternity. Despite evidence of active reproduction, ongoing bycatch remains a major threat, underscoring the urgent need for conservation actions.

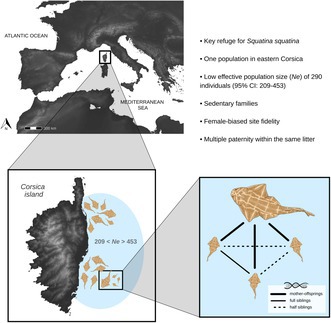

## 1 Introduction

Once common in the coastal waters of the Mediterranean, Black Sea and Northeast Atlantic, the angelshark ( *Squatina squatina* ) has disappeared from 90% of its historical geographic range over the course of the 20th century (Hiddink et al. 2019; Lawson et al. 2020; Leonetti et al. 2020; Morey et al. 2019) (Figure S1). This led to its classification as critically endangered on the Red List of the International Union for Conservation of Nature (IUCN) since 2006 (Morey et al. 2019). This widespread, almost unnoticed depletion can be attributed to the overlap between the habitat of this coastal shark and inshore fisheries (Bargnesi et al. 2022; Ellis et al. 2021), inducing overfishing (Dulvy et al. 2021), but also coastal habitat degradation and destruction. Records from the 19th and early 20th centuries indicate that angelsharks were caught using specially designed nets for their flesh and skin (Ellis et al. 2021) but were also captured as bycatch by bottom trawlers (Lawson et al. 2020). The large body size and benthic lifestyle of *S. squatina* , which lives on soft seabeds in shallow waters, render this species particularly vulnerable to accidental capture in trawls. Moreover, angelsharks possess life history traits, such as low reproductive output, lengthy pregnancy, and long generation time (Table S1), which exacerbate the impact of overfishing and slow population recovery (Dulvy et al. 2017).

Today, a few angelshark populations persist in refuges spared from intense trawling, such as the Canary Islands (Meyers et al. 2017; Meyers et al. 2024), where trawl fishing was prohibited in 1986 (Lawson et al. 2020), and Corsica (Faure et al. 2023; Lapinski and Giovos 2019), where industrial fishing pressure remains relatively low, with artisanal fisheries representing 95% of the effort (Bousquet et al. 2022; Le Manach et al. 2011). Along the eastern coast of Corsica, local artisanal fishers still recurrently catch individuals in their nets and trawls (Bousquet et al. 2024), representing the last known occurrences of the species in France, where it remains unprotected under national legislation (French Republic 1988). In other parts of the Mediterranean Sea, rare observations of angelsharks have been made recently (Bargnesi et al. 2022), such as in Sicily (Cavallaro et al. 2015), in the Adriatic Sea (Bonanomi et al. 2023; Fortibuoni et al. 2016; Holcer and Lazar 2017), in Turkey (Akyol et al. 2015), and in Tunisia (Rafrafi‐Nouira et al. 2023), reflecting the widespread local extirpations that fragmented the historical species range.

The isolation of a population, with the absence of gene exchanges, can result in a significant loss of genetic diversity across generations due to genetic drift and increased inbreeding (Frankham et al. 2002). For example, Wang et al. (2014) showed that pond frogs inhabiting isolated islands since sea level rise were deprived of inter‐island gene flow, so their genetic diversity decreased with increasing island isolation time and decreasing population size. While barriers to gene flow may be less obvious in the ocean (Benestan et al. 2021), similar patterns can be observed for marine species due, for example, to the presence of deep waters (e.g., Boussarie et al. 2022) or life cycle characteristics of some species (e.g., lack of pelagic larval dispersal; Bernardi 2000). Overfishing, among other anthropogenic selection pressures, can induce a reduction in allelic richness and heterozygosity and the disruption of spatial genetic structure (genetic homogenization) in exploited marine fish populations (Gandra et al. 2021; Pinsky and Palumbi 2014; Sadler et al. 2023).

While threatened shark species are a priority target for obtaining knowledge to inform rapid and effective conservation decisions (Hyde et al. 2022; Pacoureau et al. 2023), they are challenging to study due to the difficult access to the last populations in the vast ocean. Domingues et al. (2018) reported that only ~10% of shark and ray species have been studied for their population genetic structure, diversity, and demographic history, with 37% of these species being threatened with extinction (Dulvy et al. 2021). This lack of knowledge is even more critical for one of the most threatened chondrichthyan families, the Squatinidae (angel sharks), for which genomic population assessments are urgently needed (Ellis et al. 2021; Pearce et al. 2021). Genomic approaches are well‐suited for studying angel sharks, whose elusive nature (e.g., camouflage and scarcity) makes them rarely seen; individuals are most often available from accidental fishery catches, allowing the collection of skin samples. Genetic data can provide multiple information such as the population genetic structure (Meyers et al. 2024), relatedness between individuals (McClain et al. 2022), as well as revealing ecological and biological processes, such as philopatry and mating systems, that are otherwise difficult to study in rare marine species (Portnoy et al. 2015). While estimating the population census size (*Nc*) is challenging for angel sharks, genomic data from a subset of individuals can reveal the contemporary effective population size (*Ne*), a key concept in conservation biology (Delord et al. 2024; Hoban et al. 2020) that represents the number of breeding individuals in an ideal population (i.e., made up of diploid individuals with sexual reproduction, randomly mating, with non‐overlapping generations and no migration, mutation, nor selection) that would experience the same level of genetic drift as the natural population under study (Waples 2025). While *Nc* counts all individuals in the population, *Ne* considers only individuals actively transferring their genes to the next generation (Frankham 1995; Turner et al. 2006). By capturing recent reproductive and demographic processes and accounting for the rate of genetic diversity loss due to drift, contemporary *Ne* provides a relevant measure for assessing the vulnerability of a population to decline (Ovenden et al. 2016). Small *Ne* values indicate a high risk of inbreeding, reduced adaptive capacity, and an increased likelihood of local extinction (Clarke et al. 2024). By tracking *Ne* over time, conservationists can identify declining genetic diversity before it becomes critical, making it a key indicator of the genetic health and long‐term viability of endangered populations (Hare et al. 2011; Hoban et al. 2020; Luikart et al. 2010). Yet, this critical information is lacking for angel shark populations in the last Mediterranean refuges.

To our knowledge, only one study has investigated the population genetics of *S. squatina* (Meyers et al. 2024), focusing on individuals from the Canary Islands using both microsatellites and SNP markers. This study identified distinct genetic units, likely shaped by the oceanic depths separating the islands. Given the findings from Meyers et al. (2024), we expect the Corsican angelsharks to form a single genetic unit, as the sites sampled in our study are only separated by shallow waters. In Corsica, although recent studies have brought new knowledge about the presence and distribution of *S. squatina* (Faure et al. 2023; Lapinski and Giovos 2019) and insights into their seasonal dynamics (Bousquet et al. 2024), appropriate management and conservation of angelsharks require information on population size and structure, which have never been investigated. Here, we studied *S. squatina* individuals in Corsica to assess (i) their genetic structure, (ii) the spatio‐temporal relatedness between individuals, and (iii) their effective population size to ultimately inform conservation strategies based on population genetic health.

## 2 Materials and Methods

### 2.1 Samples Collection

**FIGURE 1:**
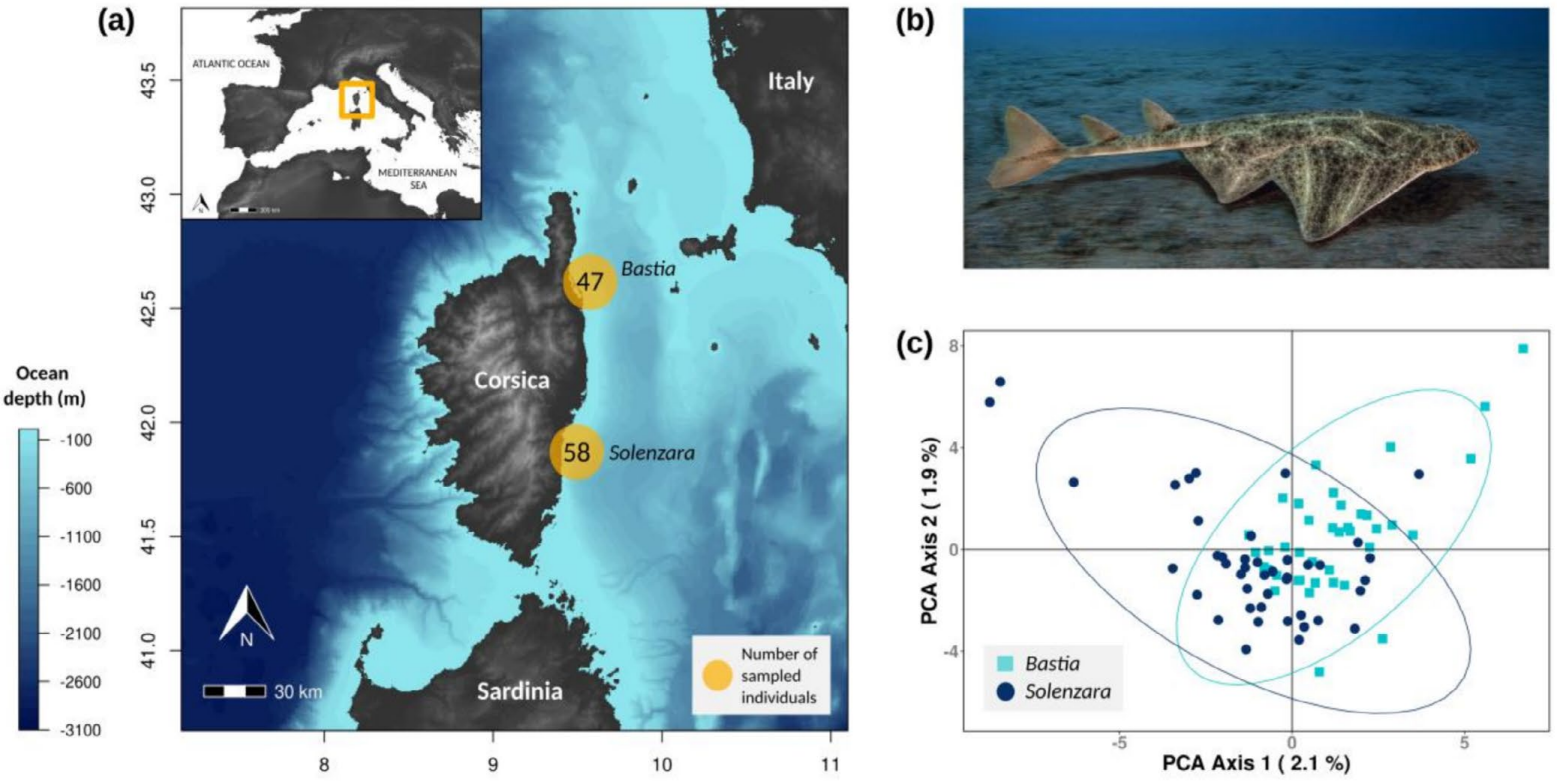
(a) Sampling map of angelshark ( *Squatina squatina* ) individual skin samples collected from bycatch by local fishers at two sites in Corsica, France: Bastia and Solenzara. (b) Angelshark observed near Bastia in 2020, on the eastern coast of Corsica. Photo credit: Laurent Ballesta. (c) Principal Component Analysis (PCA) of 83 angelshark individuals from Corsica (9138 SNPs), obtained after excluding close relatives while retaining a single representative per sibling group. The first two principal component axes describe the genetic similarity between individuals, and the ellipses represent 95% of the individuals for each sampling site (Solenzara in dark blue circles and Bastia in light blue squares), assuming a *t*‐distribution.

A total of 105 *S. squatina* individual tissues were sampled at sea between 2020 and 2022, on the east coast of Corsica (Bastia and Solenzara, Figure 1a,b). They were collected from local fishers who accidentally bycatch individuals in their trawls (Bastia), gillnets, or trammel nets (Solenzara), often at depths close to 40 m on soft bottoms. Over this two‐year period, living angelsharks were sampled during 18 opportunistic sampling events, between February and June (Figure S2), with a maximum of 30 individuals captured in one day by a single fisher in Solenzara in March 2022. Among the sampled individuals, a female gave birth to five pups aboard a fisher's boat, likely prematurely due to capture, but only three of them could be sampled. No individuals were sampled on the west coast, where the angelshark is not caught by fishers who target coralligenous reefs rather than soft bottoms. For each individual, a skin sample (1 cm^2^ fin fragment) was taken and directly preserved in pure ethanol before the release of the individual. All samples were then stored at −20°C until DNA extraction. When possible, we obtained the sampling location, the sex of the individual, its total length, and the depth of capture. Sampled individuals include 58 females, 36 males, and 11 unsexed, at various life stages (body length range: 24–120 cm). Based on their total body length (Figure S2.b), individuals were classified into three categories defined by Meyers et al. (2017): neonates: < 30 cm, pre‐adults: 30–100 cm, and adults (i.e., matures): > 100 cm.

### 2.2 DNA Extraction and SNP Genotyping

Tissue samples were sent to Diversity Arrays Technology (DArT Pty. Ltd., Canberra, Australia) for DNA extraction and genotyping‐by‐sequencing using the standard DarTseq protocol (Kilian et al. 2012; Sansaloni et al. 2011). To generate Single Nucleotide Polymorphisms (SNPs), DNA underwent a double restriction digestion. SNP calling and basic filtering were performed by DArT using a proprietary pipeline. The complete DarTseq methodology is detailed in Wells and Dale (2018). It resulted in a raw dataset containing 32,905 biallelic SNPs across 102 individuals (three individuals failed at sequencing), with a mean read depth of 7.7× and 7.33% missing data.

### 2.3 SNP Data Filtering

We imported the raw dataset into R version 4.3.3 (R Core Team 2024) for further filtered steps with the R package *dartR* (v. 2.7.2; Gruber et al. 2018; Mijangos et al. 2022) to improve data quality. A main dataset was generated using the following filtering steps: (i) SNPs with a call rate < 0.95 (i.e., > 5% missing data) were excluded, (ii) SNP reproducibility was filtered with a threshold of 0.99, (iii) a sequencing depth filter between 5× and 100× was applied, (iv) secondary SNPs were removed (i.e., only one randomly selected SNP per fragment was retained), (v) singleton SNPs (i.e., SNPs represented by a single copy in the population) were filtered out as they can be genotyping errors, (vi) SNPs potentially under strong selection were identified and excluded using the *gl.outflank* function (dartR, Whitlock and Lotterhos 2015) and (vii) monomorphic SNPs were excluded (Table S2). All individuals had a call rate > 0.90. Two individuals were excluded because they were identified as duplicates by the relatedness analysis. Data were not filtered on Hardy–Weinberg Equilibrium as it can induce the loss of valuable genetic information (Pearman et al. 2022), despite being a common practice. This resulted in a dataset of 9699 SNPs across 100 individuals (57 females, 35 males; Table S3) with a mean missing data rate of 0.67%. Across individuals, missing data ranged from 0.06% to 7.9%, with a median of 0.42%.

### 2.4 Population Genetic Structure and Diversity

Population structure was examined using three approaches. First, a Principal Component Analysis (PCA) was applied using the function *gl.pcoa* from the R package *dartR* (v. 2.7.2; Gruber et al. 2018; Mijangos et al. 2022), in order to visualize any structure present in the dataset. Second, we performed a Discriminant Analysis of Principal Components (DAPC) to identify and describe any clusters of genetically related individuals (Jombart et al. 2010), using the R package *adegenet* (v. 2.1.10; Jombart 2008). We determined the optimal number of clusters by performing a DAPC with no prior information with the function *find.clusters*. Third, we identified the optimal number of genetic clusters (K) using the *snmf* function from the *LEA* R package (v3.10.2; Frichot and François 2015), based on sparse non‐negative matrix factorization (sNMF) algorithms and cross‐entropy criteria (*K* = 1 to 8). The selected *K* value was then used to estimate individual admixture coefficients (François 2016). Additionally, genetic differentiation was determined by calculating pairwise estimates of F_ST_ (Weir and Cockerham 1984) between localities, with 10,000 bootstraps using the function *gl.fst.pop* from the package *dartR* (v. 2.7.2; Gruber et al. 2018; Mijangos et al. 2022). For comparison, genetic structure was assessed using both the full dataset (9699 SNPs across 100 individuals) and a reduced dataset in which only one representative per family group was retained (identified by the relatedness analysis, see section 2.5), resulting in 9138 SNPs across 83 individuals. This approach was motivated by the possibility that the inclusion of close relatives could bias population structure inferences, as suggested by Devloo‐Delva et al. (2019), who showed that the presence of full siblings sampled within the same year may artificially inflate signals of structure.

Genetic diversity of the population was assessed by estimating observed heterozygosity (H_O_), expected heterozygosity (H_E_), and inbreeding coefficient (F_IS_) for each potentially identified genetic cluster. The calculations were performed with the *gl.report.heterozygosity* function from the package *dartR* (v. 2.7.2; Gruber et al. 2018; Mijangos et al. 2022) and the *basic.stats* function from the R package *hierfstat* (v. 0.5‐11; Goudet 2005) to compare results.

### 2.5 Genetic Relatedness and Kinship Among Individuals

We analyzed patterns of genetic relatedness and kinship to investigate fine‐scale spatial genetic structure and potential sex‐biased patterns. Specifically, we examined whether familial relationships occurred predominantly within sites, suggesting limited dispersal, or also between sites, indicating broader mobility. Of the 100 individuals genotyped, four were related: a pregnant female captured in Solenzara and the three juveniles it gave birth to aboard a fishing boat. These individuals were used as controls to validate the calculations of relatedness and kinship estimates. To increase accuracy, we applied two different approaches, relatedness and kinship analyses, using a more stringently filtered dataset restricted to loci with complete genotyping (no missing data, 6545 SNPs across 100 individuals). First, we calculated pairwise genetic relatedness between all pairs of individuals with the Wang coefficient (J. Wang 2002), using the *coancestry* function implemented in the R package *related* (v. 1.0; Pew et al. 2015) and by adapting the approach applied in Manel et al. (2023). To assess uncertainty, we calculated 95% confidence intervals using bootstrapping over loci (500 iterations). In an ideal large random‐mating population, the expected value of relatedness is 0.5 for full siblings or parent‐offspring and 0.25 for half siblings (Table A1 in Appendix; J. Wang 2002). Additionally, we generated a Genomic Relationship Matrix (GRM) using the *gl.grm* function from the *dartR* package (Gruber et al. 2018; Mijangos et al. 2022) to estimate kinship based on an approach developed by Endelman and Jannink (2012) (Table A1; Speed and Balding 2015). Only pairs of closely related individuals (i.e., parent‐offspring, half and full siblings) identified by both methods were retained, corresponding to a Wang relatedness value ≥ 0.211 and a kinship value ≥ 0.092.

### 2.6 Contemporary Effective Population Size

We estimated the contemporary *Ne* for each identified genetic cluster(s) using the single‐sample bias‐corrected Linkage Disequilibrium (LD) method (Hill 1981; Waples 2006; Waples and Do 2010) implemented in the NeEstimator software (v.2.1; Do et al. 2014) and via the *gl.LDNe* function of the R package *dartR* (Gruber et al. 2018; Mijangos et al. 2022). As the samples used in this study include overlapping generations (i.e., age‐structured samples from 2020 to 2022), the resulting estimate from the LD method can be interpreted as the effective number of breeders that produced the cohorts represented by the samples (Waples and Do 2010). We explored *Ne* estimates using varying SNP filtering parameters (call rate by locus, Minor Allele Frequency) as recommended by Marandel et al. (2020). Singleton SNPs were removed to prevent *Ne* overestimation (Waples 2024), as a low‐stringency filter provides a balance between retaining relevant genetic information and minimizing noise (Marandel et al. 2020). We also estimated *Ne* using only mature adults (> 100 cm) to assess the impact of subadults on *Ne* calculations. *Ne* was also estimated per sampling year. Close relatives were not excluded as their removal can distort population structure by eliminating valuable genetic signals that reflect the natural composition of the population and inflate *Ne* (Waples 2025; Waples pers. comm.).

## 3 Results

### 3.1 Population Genetic Structure and Diversity

No clear genetic structure was detected among angelshark individuals from Corsica (Figure 1c). The genetic PCA shows overlapping 95% ellipses from both localities (Bastia and Solenzara), although some substructuring can be observed, particularly in 2021 (Figure S3). Similarly, the DAPC did not reveal clear clusters among individuals in eastern Corsica (Figure S4). Both sNMF and DAPC clustering algorithms identified *K* = 1 as the most likely number of clusters, corresponding to the smallest cross‐entropy criterion value (Figure S5). Nevertheless, a significant, though very low, signal of differentiation was found between Bastia and Solenzara (F_ST_ = 0.005, *p* < 0.01). After excluding related individuals (*n* = 17), leaving 83 independent individuals, results were consistent: clustering methods again favored *K* = 1, and pairwise differentiation remained low yet significant (F_ST_ = 0.004, *p* < 0.01). The DAPC was also investigated across datasets against sex, life stage, sampling month, and sampling year, to detect any pattern influenced by life events (seasonal residency, pupping) as well as potential biases related to sex; however, no structuring was observed (Figure S6).

The observed (H_O_) and expected heterozygosity (H_E_) for the whole sample size (100 individuals) was 0.115 (±0.002) and 0.118 (±0.002), respectively. When calculating for each sex, H_O_ was 0.141 (±0.002) for the 57 females and 0.161 (±0.002) for the 35 males, while H_E_ was 0.142 (±0.002) for females and 0.164 (±0.002) for males. The inbreeding coefficient (F_IS_) was 0.035 (±0.002) for the full dataset of 100 individuals and 0.033 (±0.002) when calculated using the reduced dataset without kins (9138 SNPs across 83 individuals).

### 3.2 Patterns of Family Structures

**FIGURE 2:**
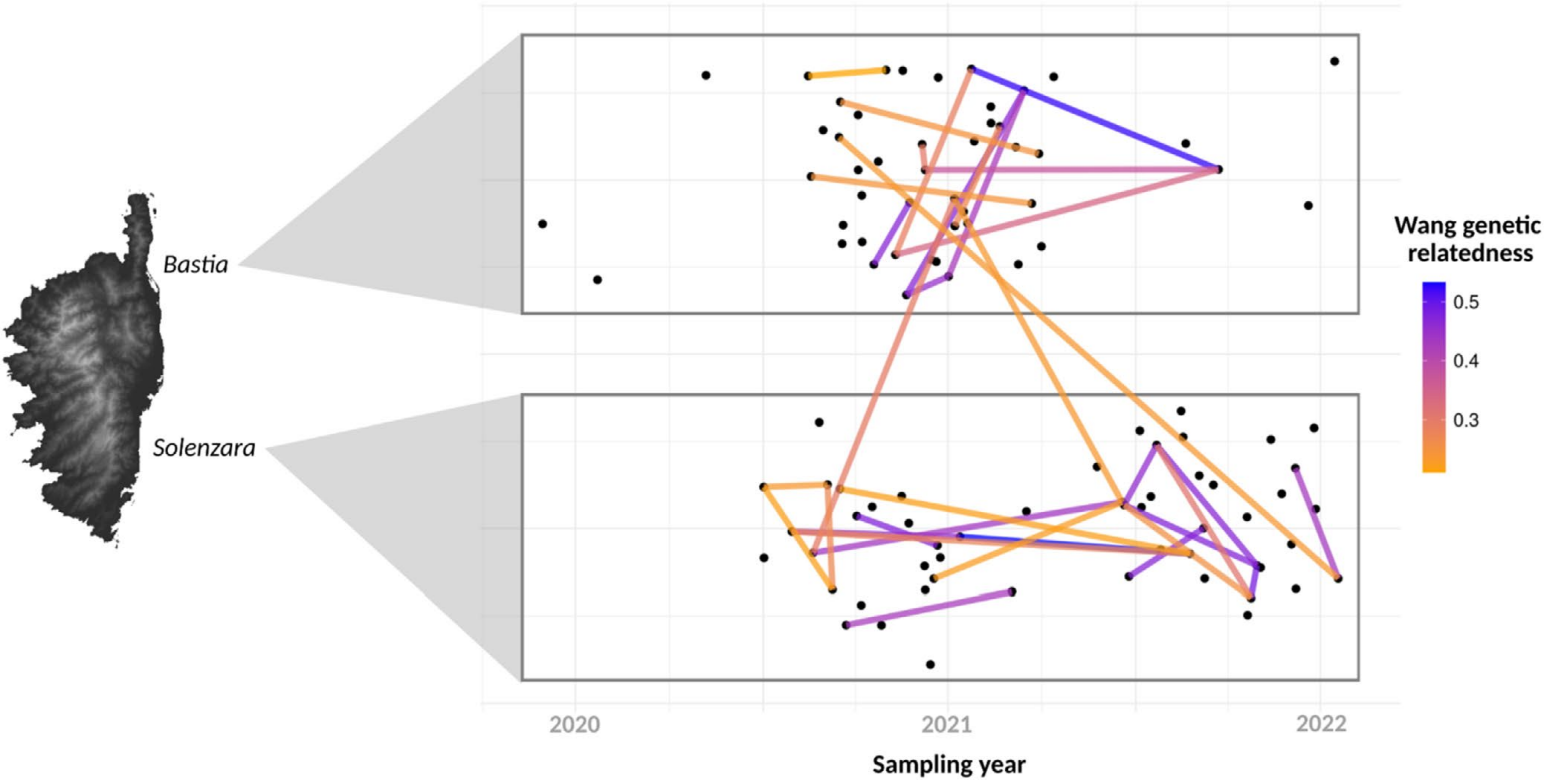
Family relationships among 35 pairs of closely related individuals, revealed by the genetic relatedness analysis (Wang coefficient) of 100 angelshark individuals (47 from Bastia, 53 from Solenzara) genotyped at 6545 SNPs. Each point represents an individual, plotted by sampling date and site (Bastia or Solenzara). Genetic relatedness between pairs ranges from 0.211 (half‐siblings) to 0.53 (full‐siblings or parent‐offspring). Points have been jittered for graphical clarity to avoid overlapping individuals sampled on the same date and location.

**FIGURE 3:**
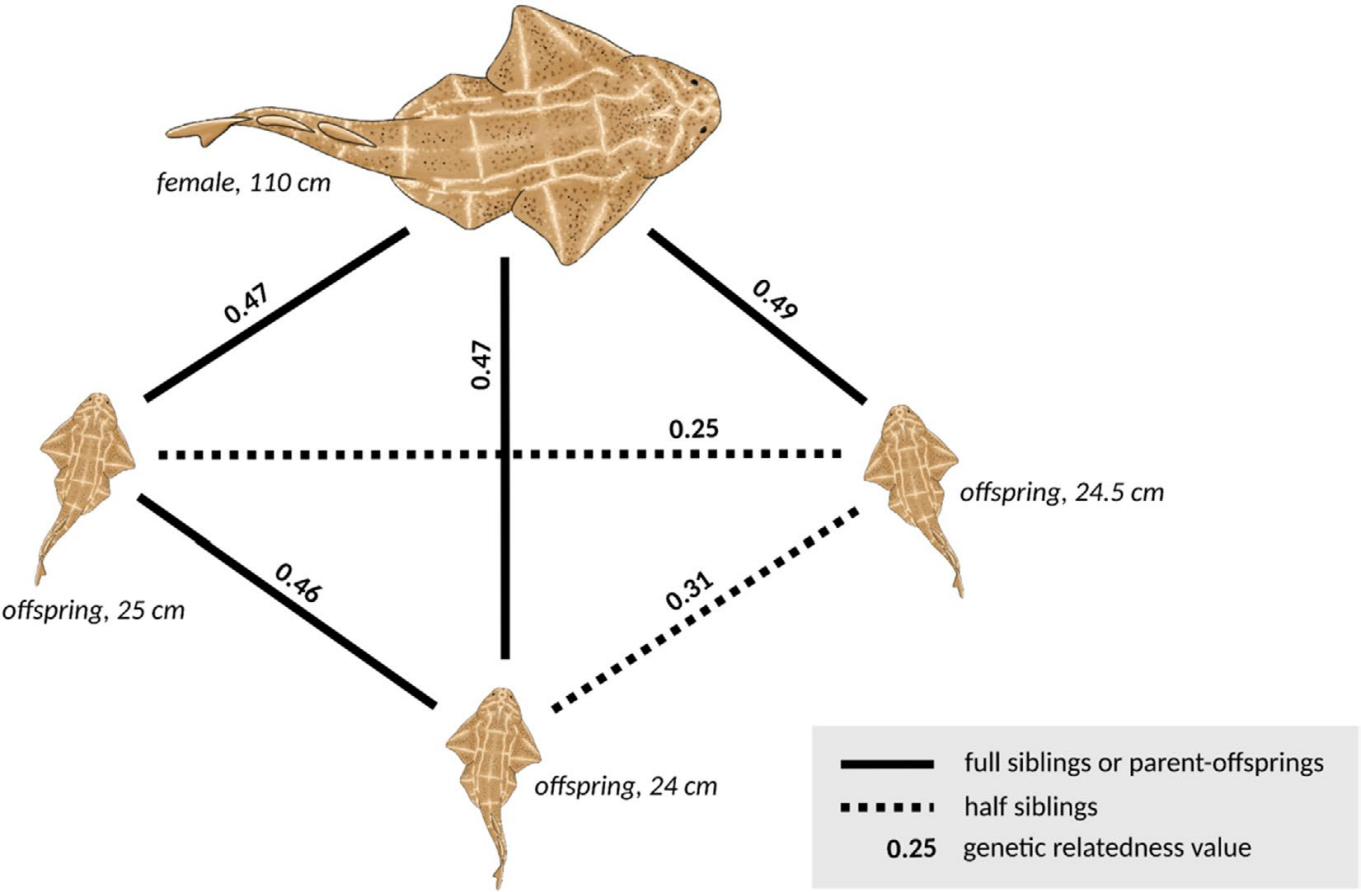
Evidence for multiple paternity revealed by genetic relatedness (Wang coefficient) among known relationships for a female angelshark ( *Squatina squatina* ) caught by fishers in Solenzara on 23/03/2022, which gave birth to three offspring on the fisher's boat. A genetic relatedness value of approximately 0.5 indicates a full‐sibling or parent‐offspring relationship, while a value of around 0.25 suggests a half‐sibling relationship. Drawing credit: Aline Faure.

**FIGURE A1:**
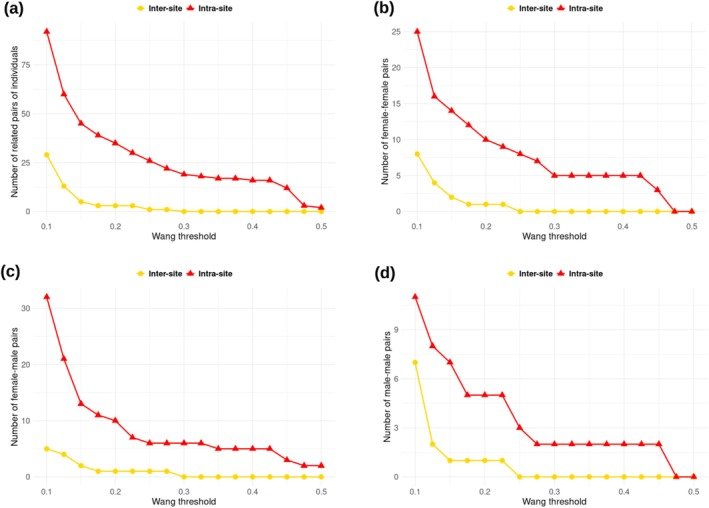
Number of related pairs of angelshark ( *Squatina squatina* ) individuals depending on the threshold chosen for the Wang relatedness value, for all possible pairs of individuals (a), for only female–female pairs (b), for only female–male pairs (c), and only male–male pairs (d). The red triangles correspond to intra‐site pairs while yellow circles correspond to inter‐site relationships.

The analysis of genetic relatedness and kinship among the 100 angelsharks identified 35 pairs of closely related individuals (i.e., parent‐offspring, half and full siblings), with genetic relatedness values ≥ 0.211 and kinship values ≥ 0.092 (Figure 2; Table S4). Two additional pairs were detected only by the Wang method and were excluded. Among the confirmed pairs, the known mother‐offspring relationship was recovered, with a mean Wang relatedness value of 0.47 (95% CI: 0.431–0.518) (Table A1), aligning with the expected 50% genetic similarity for parent‐offspring. For the three neonates born on board, the analysis revealed that two were full siblings (Wang value: 0.46), while the third one may be a half‐sibling to the others (Wang value: 0.25 and 0.31) (Figure 3). All three exhibited 100% call rate across loci. Overall, 42% of sampled individuals (42/100) were closely related to at least one other sampled individual. 20/100 individuals were strongly related in Bastia, 22/100 individuals were strongly related in Solenzara, while only 5/100 individuals had a close relative from the other site (Figure 2). When considering all possible pairs of individuals, there were consistently more intra‐site (Bastia–Bastia or Solenzara–Solenzara) than inter‐site related pairs across all Wang thresholds (Figure A1). Moreover, analyzing the influence of geographic distance on genetic relatedness revealed that closely related individuals (Wang > 0.211) are predominantly found within the same site (Figure S7). Linear regression showed a significant but weak negative effect of geographic distance on relatedness (*p* < 0.001, *R* ^2^ = 0.03), indicating a slight pattern of isolation by distance. Relatedness was significantly higher in female–female pairs compared to male–male and female–male pairs (*p* < 0.001), with male–male pairs showing the lowest relatedness overall.

### 3.3 Contemporary Effective Population Size

Because all sampled individuals belonged to a single population (see genetic structure results), and given the long generation time of angelsharks (approximately 15 years) and their low reproductive frequency (every 2 years) (Table S1), we considered individuals from two consecutive sampling years as belonging to the same cohort. Pooling across 2 years thus allowed us to increase the sample size while minimizing potential temporal biases, despite overlapping generations. This resulted in a dataset of 91 individuals (9389 SNPs; call rate across loci = 0.95 and no singletons), excluding neonates (body length < 30 cm) since they represent a new generation. Based on the body length distribution of these individuals (30–120 cm), they likely span approximately 15 different age classes, meaning the estimated *Ne* should correspond to the *Ne per generation*. These 91 individuals included 52 adults (24 females, 26 males, 2 unknown) and 39 pre‐adults (29 females, 9 males, 1 unknown) (Table S3).

The *Ne* was estimated at 290 individuals (95% CI_Jackknife_: 209–453). By varying the SNP filtering parameters (call rate and MAF thresholds), this value was lower with stringent SNP filtering than with low‐stringent SNP filtering (Table S5). Excluding immature individuals did not drastically change the *Ne* value, which was estimated to be 329 (95% CI_Jackknife_: 189–1102), but with a larger confidence interval due to the smaller sample size (Table S6). When estimated for each year separately, *Ne* was estimated at 230 (95% CI_Jackknife_: 156–419) in 2021 and 176 (95% CI_Jackknife_: 84–∞) for angelsharks sampled in 2022. However, this difference between years is biased by the unbalanced number of sampled individuals (Table S7).

**FIGURE 4:**
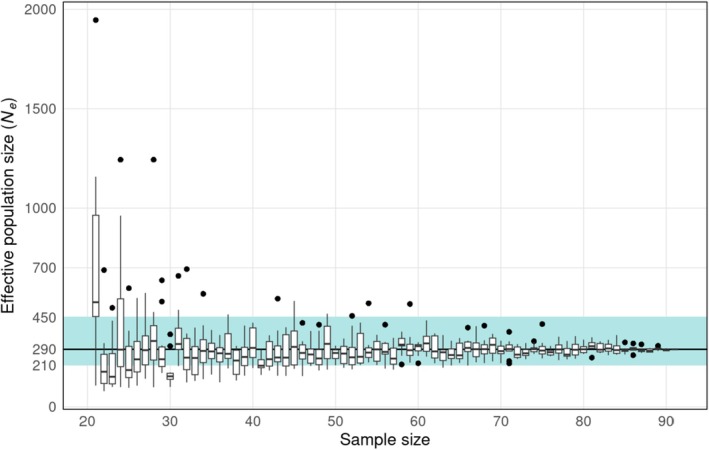
Influence of sample size (number of angelshark individuals sampled) on the precision of the effective population size (*Ne*) estimate. For each sample size, the *Ne* was calculated 10 times using randomly selected individuals. The black line represents the *Ne* value of 290, calculated with 91 individuals. The blue shaded area shows the 95% confidence interval for *Ne* = 290 (210–450), estimated using the Jackknife method for the sample size of 91 individuals. See Figure S8 for complete data on sample sizes ranging from 5 to 91.

To study the influence of the sample size on *Ne* estimation, we generated subgroups of randomly selected individuals over the 2021–2022 period, with sample sizes ranging from 5 to 91 individuals, for which we calculated *Ne* with 10 iterations. The results show that the precision and accuracy of *Ne* estimates increase with the number of sampled individuals (Figure 4). For small sample sizes, there is a large variance in *Ne* estimates, which decreases as sample size increases (Figure S8), and with the presence of *Ne* “outliers”. The estimation of *Ne* becomes relatively accurate from 60 randomly sampled individuals over the 2021–2022 period (Figure 4). With fewer than 60 individuals, some estimated *Ne* values are outside the 95% confidence interval estimated by the JackKnife method from the sample of 91 individuals.

## 4 Discussion

### 4.1 Population Genetic Structure and Diversity

Overall, our structure analyses indicate a connected angelshark population along the east coast of Corsica, while highlighting that subtle genetic differentiation cannot be entirely excluded. A weak but significant differentiation between Bastia and Solenzara supports this possibility, and the signal persisted even after removing close relatives, suggesting it is not an artifact of kin sampling (Devloo‐Delva et al. 2019).

In the Canary Islands, Meyers et al. (2024) found that the dispersal of *S. squatina* is likely constrained by ocean depth, a pattern also observed in the Pacific angelshark ( *S. californica* ) by Gaida (1997). This finding is also supported by isolation‐by‐resistance models in the gray reef shark, a reef‐associated species in the tropical Indo‐Pacific, where Boussarie et al. (2022) showed that deep oceanic waters act as a strong barrier to dispersal, while proximity to habitat facilitates movement. In Corsica, the continuous shallow waters along the east coast likely promote connectivity between Bastia and Solenzara. It would be interesting to investigate whether angelsharks are also present along the west coast of Corsica, despite the lack of reported observations by fishers (only one female was reported in May 1988 in Nonza by Riutort 1989), and to assess whether they constitute a distinct population from that of the east coast. Given that *S. squatina* generally occurs at depths < 150 m, the relatively shallow waters between Corsica and Sardinia (Figure 1a) likely facilitate exchanges between these two islands, supporting the idea of a historically connected population. In contrast, potential exchanges with the Tuscan Archipelago and mainland Italy are less certain, as individuals would need to cross deeper waters (350–450 m), where angelshark presence is rare. Further research on depth use is needed to better assess their dispersal potential and connectivity with the Italian mainland and islands.

The heterozygosity values observed in Corsican angelsharks (H_O_ = 0.115, H_E_ = 0.118) were relatively low compared to estimates reported for other shark species also genotyped with DArT SNPs. For instance, H_E_ ranged from 0.129–0.140 in great hammerhead sharks ( *Sphyrna mokarran* ; Brunjes et al. 2024), 0.211–0.214 in Galapagos sharks ( *Carcharhinus galapagensis* ; Pazmiño et al. 2017), and 0.269–0.285 in gray nurse (*Carcharias taurus*; Reid‐Anderson et al. 2019) and school sharks ( *Galeorhinus galeus* ; Devloo‐Delva et al. 2019). For a better comparison, sequencing museum samples of *S. squatina* would allow assessment of historical and contemporary genetic diversity, helping to determine whether angelsharks have suffered a decline over the past decades. A reduced diversity may reflect a small population size and limited gene flow, possibly due to the loss of surrounding populations through overfishing. Genetic diversity can also be strongly influenced by reproductive strategies (Domingues et al. 2018), such as parthenogenesis, multiple paternity, and philopatry.

### 4.2 Patterns of Site Fidelity

Of the 35 pairs of closely related individuals (Figure 2, Table S4), only three pairs were detected between Bastia and Solenzara (approximately 100 km), supporting dispersal along the eastern Corsican coast. All other pairs were within sites, indicating site fidelity. The significantly higher relatedness among female–female pairs compared to male–male and male–female pairs suggests sex‐biased dispersal. Females may remain closer to, or return to, their natal sites for reproduction, which would favor the formation of kin groups within sites. Such female philopatry could help explain the weak but significant differentiation detected between Bastia and Solenzara. In contrast, males may disperse more widely, thereby reducing relatedness among males and contributing to gene flow between localities. This interpretation is consistent with observations in the Canary Islands, where telemetry has shown that male angelsharks are generally more mobile than females (Mead et al. 2023) and spend time in shallow habitats predominantly in winter, hypothesized to be the mating season (Meyers et al. 2017). In Corsica, fishers observe angelsharks throughout the year, with notably large aggregations of mature angelsharks at the end of winter (Bousquet et al. 2024). Reproductive philopatry (i.e., breeding‐site fidelity) has never been described in angelsharks, but this behavior is commonly observed in chondrichthyans (Chapman et al. 2015; Flowers et al. 2016).

### 4.3 Evidence for Multiple Paternity

Genetic relatedness analysis revealed half‐siblings among the three known offspring born on the fisher's boat from the same mother (Figure 3). Comparable values for half‐sibling pairs (0.22–0.34) have been reported by Nevatte et al. (2023) within a seven‐pup litter of sawsharks ( *Pristiophorus cirratus* ), supporting the reliability of our estimates. While multiple paternity is well documented in many shark species (e.g., DiBattista et al. 2008; Larson et al. 2011; Nevatte et al. 2023; Rossouw et al. 2016), it has never been reported in *S. squatina* . This suggestive evidence of multiple paternity in *S. squatina* may align with the species' tendency to aggregate in coastal shallow waters during the mating season (Meyers et al. 2017). Similarly, the aggregative behavior of the common smooth‐hound ( *Mustelus mustelus* ) has been associated with its relatively high occurrence of polyandry (Rossouw et al. 2016). In our case, the evidence for multiple paternity in *S. squatina* is preliminary, as it is based on a single litter of only three pups. Further confirmation from additional litters is needed, but this discovery contributes to understanding the reproductive strategies of this critically endangered species and raises important questions about the potential role of coastal shallow waters as key areas for its reproduction.

### 4.4 Contemporary Effective Population Size

For a simplified conservation point of view, *Ne* can be interpreted as the average number of breeding individuals that produced the cohorts encompassed by sampling (2021–2022). A *Ne* of 290 individuals suggests that the population genetic diversity behaves as if it were inherited from 290 breeding individuals. While the census size may represent thousands of individuals (considering the regular bycatch of hundreds of individuals within just a few days), the relatively low *Ne* likely reflects limited genetic diversity. This may be exacerbated by the localized reproduction of individuals, as suggested by the presence of family structure predominantly found at each site. A potential genetic bottleneck caused by intensive fishing during the 20th century could also contribute to this low estimate, though its genetic effects may be delayed and not yet detectable given the species' long generation time (Gargiulo et al. 2025). The lower fishing pressure and habitat degradation in Corsica (Le Manach et al. 2011; Bousquet et al. 2022) compared to the mainland are likely to have allowed angelsharks to persist, as observed by Corsican fishers who have never seen the angelshark disappear on their island. Yet, given the low *Ne*, the long‐term absence of genetic connectivity to other populations would be detrimental to this population. In isolated populations, genetic variation can take a very long time to be reestablished through mutation (Hare et al. 2011), while gene flow (even at very low levels of 1 effective individual per generation) can alleviate inbreeding depression (Waples 2024) and reduce extinction risk. Future studies should investigate the angelsharks' genetic structure across their range. Comparing local and global *Ne* (Hare et al. 2011) could help determine whether the Corsican population is isolated, with no episodic gene flow, or if it is part of a metapopulation with other remaining populations.

To our knowledge, no contemporary estimates of *Ne* are currently available for other *Squatina* species. We therefore compare the LD‐based *Ne* obtained for Corsican angelsharks (290; 95% CI: 209–453) with values reported for other elasmobranch species using the same method (listed in Table A2). This estimate is relatively low compared to species with similar life history traits (long gestation period, low fecundity, late maturity), such as the sandbar shark ( *Carcharhinus plumbeus* , *Ne* = 3977; Portnoy et al. 2009) and the white shark ( *Carcharodon carcharias* , *Ne* = 1512; Blower et al. 2012). However, it is closer to values reported for critically endangered elasmobranch species such as the smalltooth sawfish ( *Pristis pectinata* , *Ne* = 295; Chapman et al. 2011) and the scalloped hammerhead shark ( *Sphyrna lewini* , *Ne* = 307; Pinhal et al. 2020), both experiencing population declines due to overfishing, further exacerbated by their aggregative behavior in coastal waters. Conversely, some other critically endangered species, such as the blue skate ( *Dipturus batis* , *Ne* = 21,015; Delaval et al. 2022) and the blue shark ( *Prionace glauca* , *Ne* = 850; Dolfo et al. 2024), exhibit much higher *Ne* values, likely reflecting their life history traits, including higher fecundity and broader dispersal. Future research should explore whether coastal elasmobranchs with slow life history traits have always had naturally small *Ne* or whether their *Ne* has declined over time due to anthropogenic pressures.

### 4.5 Sampling Effort for Reliable Effective Population Size Estimates

Increasing the number of individuals has been shown to result in more accurate estimates of *Ne* (Luikart et al. 2010; Nunziata and Weisrock 2018), but this requires more sampling effort. While highlighting the importance of sampling a large number of individuals, our results indicate that approximately 60 individuals are sufficient (in the case of this angelshark population) to reliably estimate *Ne* with the LD method (Figure 4). This would reduce the time and cost of sampling and sequencing for future monitoring of angelshark populations. It would be interesting to estimate again the *Ne* in 10 years' time, for example, considering the angelshark generation length of about 15 years, by sampling > 60 individuals along the east coast of Corsica. By comparing the obtained *Ne* value with that of this study (ensuring a similar composition of individuals in terms of ages and sex ratio, a similar number of SNPs and missing data percentage, and applying the same SNP filtering parameters), it would help to track changes and monitor the population's genetic health.

### 4.6 Conservation Implications

Although the presence of newborns and gravid females indicates that the Corsican angelshark population is reproductively active, the *Ne* estimated between 209 and 453 individuals is below the traditionally recommended threshold of 500 (Franklin 1980), which has been considered necessary to minimize inbreeding depression and demographic stochasticity effects (Palstra and Ruzzante 2008). Frankham et al. (2014) even highlighted that a *Ne* ≥ 1000 would be needed for preserving long‐term adaptive potential. However, these thresholds may not be universally applicable and should be more nuanced by considering species‐specific life history traits and ecological contexts to effectively assess genetic health (Clarke et al. 2024; Kimble et al. 2023). This low *Ne* is concerning, especially considering that this population may not be connected to others by migration and also because Corsican fishers sometimes bycatch more than a hundred individuals in less than two days. Angelsharks are then released at sea, as required by the European Council Regulation No. 2019/1241, which prohibits EU vessels from fishing, retaining on board, transhipping, and landing this species (Morey et al. 2019), but some individuals are found dead in fishing areas due to traumatic entanglement in nets. Bousquet et al. (2024) estimated a mortality of 27% among fished angelsharks observed during their small‐scale fisheries monitoring campaigns in Corsica. The sedentary nature of angelsharks, evidenced by the presence of family structures restricted to specific sites, heightens their vulnerability to fishing bycatch. This finding aligns with the recommendation made by the Scientific, Technical and Economic Committee for Fisheries in 2003 concluding that *S. squatina* should be managed on the smallest possible spatial scale (Ellis et al. 2021).

The capture of 30 angelshark individuals by a fisher in a single day in March, at a depth of 35 m in Solenzara waters, coincided with aggregations of picarels ( *Spicara smaris* ) which form large breeding colonies made of nests on sandy seabeds at the beginning of spring (Deter et al. 2024). These ephemeral aggregations attract a diverse array of predators, including predatory fish (e.g., *Zeus faber*), elasmobranchs that feed on picarels, and local fishers targeting *Z. faber* and capturing elasmobranchs as bycatch. At a picarel arena site on the central east coast, local fishers even reported bycatching approximately 300 angelsharks in their trawls over 2 days, including numerous gravid females. The preservation of these functional zones of high fisheries interest is crucial for the survival of numerous IUCN Red List threatened elasmobranch species (*S. squatina, Rostroraja alba, Raja clavata, Squalus blainville, Mustelus mustelus, Dasyatis pastinaca*), as well as commercially important species like *Z. faber* . Further research is needed to assess the ecological importance of these seasonal prey concentrations for elasmobranchs, which may rely on such temporary hotspots for feeding. Understanding the spatiotemporal relationship between picarel breeding colonies and the angelshark life cycle would help identify Critical Angel Shark Areas (CASAs; Gordon et al. 2019) to ensure the long‐term survival of the population. Implementing seasonal fishing restrictions in such sensitive areas could mitigate bycatch of this threatened species, along with other top‐predator species that play a vital ecological role in marine ecosystems (Dulvy et al. 2024).

To facilitate the recovery of angelshark populations, we recommend adding *S. squatina* to the list of protected species in all countries where it has been present, alongside modifications to fishing practices. While the implementation of effective selective gear is challenging given the size and morphology of angelsharks, measures such as enforcing seasonal closures in critical areas (e.g., picarel breeding colonies in March in Corsica but often late elsewhere) could greatly reduce bycatch and contribute to population restoration. Additionally, preserving the integrity and ecological function of estuaries and shallow coastal waters—key habitats for angelshark parturition and juvenile development—is essential, particularly considering the species' low reproductive capacity and slow recovery rate. The successful rebound of angelshark populations is possible if conservation strategies are grounded in a robust understanding of the species' biology, local ecology, and population dynamics.

Corsica was recently designated an Important Shark and Ray Area (ISRA; Jabado et al. 2023; Hyde et al. 2022), underscoring its unique role as a refuge for vulnerable elasmobranchs (Pichot et al. 2024). However, no concrete conservation measures have been implemented so far. The present study provides valuable insights that should be integrated into the revision of regional conservation action plans (Gordon et al. 2019). Ensuring the long‐term viability of the angelshark population in this last northwestern Mediterranean refuge is of paramount importance, as it may serve as a source population for recolonizing Italian islands, where angelsharks were once abundant but have now nearly disappeared.

## Author Contributions

**Nadia Faure:** conceptualization (equal), data curation (lead), formal analysis (lead), investigation (lead), methodology (equal), visualization (lead), writing – original draft (lead), writing – review and editing (equal). **Maurine Vilcot:** formal analysis (supporting), methodology (supporting), writing – review and editing (equal). **Franck Pichot:** resources (equal), writing – review and editing (equal). **Jean‐Jacques Riutort:** resources (equal), writing – review and editing (equal). **Adèle Barroil:** resources (equal), writing – review and editing (equal). **Florian Holon:** resources (equal), writing – review and editing (equal). **Nicolas Tomasi:** resources (equal), writing – review and editing (equal). **David Mouillot:** conceptualization (supporting), funding acquisition (supporting), writing – review and editing (equal). **Julie Deter:** conceptualization (equal), funding acquisition (lead), project administration (lead), supervision (equal), writing – review and editing (equal). **Stéphanie Manel:** conceptualization (equal), methodology (supporting), project administration (equal), supervision (equal), writing – review and editing (equal).

## Conflicts of Interest

The authors declare no conflicts of interest.

## Supporting information


**Data S1:** ece372275‐sup‐0001‐Supinfo.pdf.

## Data Availability

The R scripts are available at https://gitlab.mbb.cnrs.fr/nfaure/angelshark‐population‐genetics.git, while metadata and raw SNP data generated for this study are available on the Zenodo repository via the following link for the peer review process: https://doi.org/10.5281/zenodo.15304015.

## Appendix A

**TABLE A1:** Genetic relatedness and kinship values and 95% confidence intervals for pairs of angelshark ( *Squatina squatina* ) individuals with known relationships, compared with the reference values. The Wang estimator quantifies the proportion of alleles that two individuals share due to common ancestry, by comparing the observed sharing of alleles at multiple loci with their expected sharing based on population allele frequencies. Rather than measuring the total proportion of shared alleles, as in genetic relatedness, kinship quantifies the probability that a randomly selected allele from one individual is identical by descent (i.e., inherited from a shared ancestor) with a randomly selected allele from another individual. Since each individual possesses two alleles per locus, and kinship calculations consider only one randomly drawn allele at a time, the resulting kinship values correspond to half of the relatedness coefficient.

Relationship	*Genetic relatedness (Wang coefficient)*		*Kinship (Genomic Relationship Matrix method)*	
	*S. squatina*	Reference value (J. Wang 2002)	*S. squatina*	Reference value (Speed and Balding 2015)
Clones	0.935^a^ [0.885, 1]	1	0.47^a^	0.5
Parent‐Offspring	0.474^b^ [0.431, 0.518]	0.5	0.22^b^	0.25 [0.204, 0.296]
Full‐siblings	0.457^c^ [0.422, 0.497]	0.5	0.216^c^	0.25 [0.204, 0.296]
Half‐siblings	0.281^d^ [0.211, 0.353]	0.25	0.138^d^	0.125 [0.092, 0.158]
First cousins	Unknown	0.125	Unknown	0.062 [0.038, 0.089]
Unrelated	0	0	0	0

^a^ Average of the 30 pairs of individuals duplicated during sequencing.

^b^ Average obtained from values between the mother (NF176) and its 3 offspring (NF87, NF100, NF101).

^c^ Value obtained for full‐sibling relationships between offsprings NF100 and NF101.

^d^ Average obtained from values between presumed half‐siblings (offsprings NF087‐NF100 and NF087‐NF101).

**TABLE A2:** Effective population size (*Ne*) estimates and 95% confidence intervals (CI) from other studies on elasmobranch species, using the linkage disequilibrium (LD) method.

References	Species	Population location	IUCN Red List category^a^	Ecology and life history traits	Contemporary LD *Ne*	95% CI for *Ne*	Pcrit^b^	Number of individuals used	Number of genetic markers	Interpretation of *Ne* by its authors
Present study: Faure et al. (2025)	Angelshark ( *Squatina squatina* )	Corsica island	Critically Endangered	Coastal. Lecithotrophic viviparity. Biennial reproduction. Low fecundity (< 20). Generation time may be 15 years.	290	209–453 (Jackknife)	singletons	91	9389 SNPs	Low *Ne* which may reflect the effects of overfishing.
Portnoy et al. (2009)	Sandbar shark ( *Carcharhinus plumbeus* )	Lagoons of Delaware Bay	Vulnerable	Coastal. Long gestation period. Low fecundity. Generation time ~20 years.	3977	1900‐∞	0.01	396	8 microsatellite loci	The species' evolutionary potential should not be compromised
Chapman et al. (2011)	Smalltooth sawfish ( *Pristis pectinata* )	Florida coastal waters	Critically Endangered	Coastal. Generation time may be 27 years.	295	170–901	0.01	72	8 microsatellite loci	*Ne* is modest.
Blower et al. (2012)	White shark ( *Carcharodon carcharias* )	Pelagic Australian waters	Vulnerable	Pelagic. Site fidelity. Late maturity (7–17 years). Low fecundity (2–12). Infrequent reproduction. Longevity 60 years.	1512	122‐∞	0.06	97	6 microsatellite loci	Low and close to thresholds (*Ne* = 1000) at which adaptive potential may be lost.
Dudgeon and Ovenden (2015)	Zebra shark ( *Stegostoma fasciatum* )	Eastern Australian coasts	Vulnerable	Coastal. Maturity at 6 years. Oviparous species. Fecundity 80 eggs. Longevity 28 years.	377	256–677 (Jackknife)	0.01	114	13 microsatellite loci	Low *Ne* may be indicative of a healthy population of a relatively rare species
Pazmiño et al. (2017)	Galapagos shark ( *Carcharhinus galapagensis* )	Galapagos islands	Near Threatened	Coastal. Late maturity (10 years). Gestation period 12 months. Low fecundity.	205	NA	0.02	54	7934 SNPs	Low *Ne*
Reid‐Anderson et al. (2019)	Gray nurse shark ( *Carcharias taurus* )	Eastern Australian coasts	Critically Endangered (Australia)	Coastal. Sexual maturity 6–12 years. Low fecundity (2 pups every 2 years). Longevity ~40 years.	416	403–428	0.02	57	3087 SNPs	Forward simulations demonstrated that a *Ne* of 400 will result in a loss of genetic variation through genetic drift over the next 50 generations.
Pinhal et al. (2020)	Scalloped hammerhead shark ( *Sphyrna lewini* )	Brazilian waters	Critically Endangered	Coastal and semi‐oceanic. Migratory. Late maturity. Long gestation period. Low reproductive capacity.	307	203–553	0.05	229	10 microsatellite loci	Low *Ne* highlights the need for urgent conservation measures to safeguard the demographic and evolutionary potential of this endangered species
Delaval et al. (2022)	Blue skate ( *Dipturus batis* )	British Shelf, North‐East Atlantic	Critically Endangered	Demersal offshore, also found in coastal waters. Oviparous with ~40 egg‐cases deposited annually.	21,015	17,110–27,213 (Jackknife)	0.02	406	6350 SNPs	Relatively high *Ne* compared to other *D. batis* populations.
Liu et al. (2023)	Sicklefin lemon shark ( *Negaprion acutidens* )	Dongsha Island, China	Endangered	Coastal. Strong site fidelity. Female philopatric behavior. Placentally viviparous, producing 1–13 pups per litter.	86	75–100	0.02	188 juveniles	9 microsatellite loci	Insufficient for short‐term and long‐term health of this species
Dolfo et al. (2024)	Blue shark ( *Prionace glauca* )	Mediterranean pelagic waters	Critically Endangered	Pelagic and migratory. Early maturity (4–6 years). High fecundity (30 pups). Generation time 8 years.	850	450–1840	0.02	167	22 microsatellite loci	Fivefold lower than *Ne* of the Atlantic population (*Ne* = 4500)

^a^ At the time of each publication.

^b^ Pcrit = rare‐allele screening critical value, i.e., Minor Allele Frequency (MAF).
