# Supplementary material for: A Fragile Stronghold: Genomics Reveal Angelshark Population Vulnerability in Corsica, a Key Mediterranean Refuge
Source: Ecol Evol. 2025 Oct 13;15(10):e72275. doi: 10.1002/ece3.72275 (PMC12516160; doi:10.1002/ece3.72275)
Supplement: Supplementary file 1 — Data S1: ece372275‐sup‐0001‐Supinfo.pdf. [file ECE3-15-e72275-s001.pdf]

Supporting Information

Supporting tables

Table S1. Life history traits for the angelshark, *Squatina squatina* (from Ellis *et al*. 2020 and Lawson *et al.* 2020)

**Life history traits**	**Angelshark (*Squatina squatina*)**	**References**
Generation length	14.5 years for *Squatina californica.* Unknown for *S.squatina*	Cailliet *et al.* (1992)
Growth rate	Overall growth rate of 8.86 cm per year (captive individual)	Cavallaro *et al.* (2015)
Frequency of reproduction	Probably biennial reproductive cycle	Capapé *et al.* (1990); Baremore (2010)
Gestation period	6-12 months	Awruch *et al.* (2008)
Fecundity	Usually less than 20 pups per female	Ellis *et al*. (2020)
Maximum body size	244 cm	Compagno *et al*. (1984)
Body size at maturity	>100 cm	Bousquet *et al*. (2024)
Body length at birth	24-34 cm	Lo Bianco (1899); Capapé *et al.* (1990)
Sexual dimorphism	Females larger than males	Capapé *et al.* (1990)
Reproductive mode	Lecithotrophic viviparous (with yolk sacs)	Capapé *et al*. (1990); Osaer *et al.* (2015)
Longevity	Unknown	/

Table S2. Eight filtering steps of SNPs and angelshark (*Squatina squatina*) individuals to create the main dataset.

Filtering step	Number of SNPs	Number of individuals	Percentage of missing data
1. Initial raw dataset	32,905	102[a]	7.33
2. Filter call rate by locus (0.95)	22,175	102	1.06
3. Filter SNP reproducibility (0.99)	11,463	102	0.93
4. Filter read depth (5-100)	10,128	102	0.68
5. Filter secondaries (keep 1 SNP per fragment)	9,808	102	0.67
6. Filter singleton alleles (1/2N)[b]	9,779	102	0.67
7. Filter loci under selection (outflank)	9,774	102	0.67
8. Filter duplicate individuals	9,699	100	0.67

[a]Of the 105 individuals sampled, 3 individuals failed at sequencing

[b] MAF threshold equals to approximately 0.005

Table S3. Age structure and sex for the 100 sequenced angelsharks sampled in Bastia and Solenzara, Corsica, between 2020 and 2022. Individuals were classified into three body length categories defined by Meyers *et al.* (2017): neonates: <30 cm, pre-adults: 30-100 cm and adults: >100 cm.

		2020	2021	2022	**Total**
Adults	Bastia	0	13 females<br>8 males<br>2 unknown	2 males	**52**
	Solenzara	0	3 females<br>3 males	8 females<br>13 males	
Pre-adults	Bastia	0	13 females<br>4 males<br>1 unknown	1 female<br>1 male	**39**
	Solenzara	0	11 females<br>3 males	4 females<br>1 male	
Neonates	Bastia	0	0	0	**7**
	Solenzara	0	4 females	3 unknown	
NA	Solenzara	2 unknown	0	0	**2**
**Total**		**2**	**65**	**33**	**100**

Table S4. Thirty-five pairs of *S. squatina* individuals having a Wang genetic relatedness value ≥0.211 and a kinship value ≥ 0.092 (i.e. half, full siblings and parent-offsprings), ranked from higher to lower Wang values. The pairs of individuals with a known mother-offspring or offspring-offspring relationship are shown in red bold italics. The pairs formed between angelsharks from different sampling sites are indicated in blue bold. The table also indicates the sampling site of each individual (BB=Bastia-Bastia, BS=Bastia-Solenzara, SS=Solenzara-Solenzara), the sex of each individual (F=Female, M=Male, U=Unknown) and the Euclidean distance in kilometres between individuals.

Individual 1	Individual 2	Wang	Wang low 95% CI	Wang high 95% CI	Kinship	Distance (km)	Site	Sex
NF053	NF097	0.5326	0.5047	0.5602	0.2737	0	SS	MF
NF003	NF021	0.5246	0.4901	0.5616	0.221	4.6107	BB	MF
NF087	***NF176***	***0.4849***	***0.4555***	***0.5181***	***0.2324***	***0***	***SS***	***UF***
NF066	NF185	0.4737	0.4361	0.5114	0.2357	0.027	SS	MM
NF100	***NF176***	***0.4724***	***0.4388***	***0.5005***	***0.2138***	***0***	***SS***	***UF***
NF116	NF175	0.4682	0.4318	0.5104	0.2621	0	SS	MM
NF024	NF041	0.4681	0.4306	0.5077	0.2618	0	BB	FF
NF028	NF113	0.4659	0.421	0.5041	0.2366	0	BB	FF
NF101	***NF176***	***0.4659***	***0.4314***	***0.4955***	***0.2144***	***0***	***SS***	***UF***
NF100	***NF101***	***0.4572***	***0.4217***	***0.4971***	***0.2162***	***0***	***SS***	***UU***
NF053	NF184	0.4543	0.4223	0.4858	0.2167	0.098	SS	MF
NF023	NF113	0.4504	0.4089	0.492	0.1992	0	BB	FF
NF063	NF115	0.4356	0.3926	0.4756	0.2321	0.098	SS	FM
NF076	NF171	0.4307	0.3955	0.4638	0.2253	0	SS	MF
NF023	NF028	0.4274	0.3845	0.4756	0.2101	0	BB	FF
NF074	NF075	0.4269	0.3919	0.4647	0.2161	0	SS	FF
NF003	NF005	0.3758	0.333	0.4175	0.1319	0.391	BB	MU
NF003	NF163	0.3387	0.2952	0.378	0.1168	4.611	BB	MF
NF087	***NF101***	***0.3081***	***0.2605***	***0.3532***	***0.1536***	***0***	***SS***	***UU***
NF005	NF033	0.2976	0.2568	0.3399	0.1117	4.446	BB	UF
**NF043**	**NF063**	**0.2946**	**0.2513**	**0.3385**	**0.1517**	**66.282**	**BS**	**MF**
NF021	NF163	0.2896	0.2451	0.331	0.1046	0	BB	FF
NF097	NF184	0.2779	0.2319	0.3226	0.1397	0.098	SS	FF
NF018	NF031	0.2716	0.2242	0.3127	0.1373	0	BB	MM
NF072	NF054	0.271	0.226	0.3176	0.1307	12.420	SS	FF
NF087	***NF100***	***0.2537***	***0.211***	***0.2995***	***0.1222***	***0***	***SS***	***UU***
NF027	NF164	0.2478	0.1994	0.295	0.1298	0	BB	FF
NF015	NF032	0.2453	0.1975	0.2894	0.1360	0	BB	MM
**NF043**	**NF115**	**0.2373**	**0.1885**	**0.2841**	**0.1336**	**66.335**	**BS**	**MM**
NF072	NF186	0.2365	0.19	0.2755	0.1116	12.420	SS	FM
**NF025**	**NF171**	**0.236**	**0.1901**	**0.2845**	**0.1278**	**65.643**	**BS**	**FF**
NF114	NF115	0.2257	0.1724	0.27	0.1371	0	SS	MM
NF054	NF186	0.2211	0.172	0.2625	0.1156	0	SS	FM
NF061	NF097	0.2208	0.1796	0.2673	0.1291	0.098	SS	MF
NF013	NF037	0.212	0.1648	0.2612	0.1121	0	BB	FF

Table S5. Estimates of the effective size (*Ne*) and 95% confidence interval (CI) for the angelshark (*S. squatina*) population in Corsica, using the Linkage Disequilibrium method, with different SNP filtering parameters.

** excluding alleles represented by a single copy in the population*

Sample size	Call rate across loci	MAF threshold	*Ne*	Lower JackKnife 95% CI	Upper JackKnife 95% CI
100	0.7	All alleles	349.4	281	457
		No singletons*	301	222.5	452.3
		0.01	301	222.5	452.3
		0.02	248.8	181.3	382.2
		0.05	235.7	169.1	371.8
	0.9	All alleles	353.8	285.5	460.5
		No singletons*	297.6	220	447
		0.01	297.6	220	447
		0.02	252.5	183.3	390.4
		0.05	243	174.3	383.8
	0.95	All alleles	357	287.5	466.3
		No singletons*	296.6	218.3	448.9
		0.01	296.6	218.3	448.9
		0.02	253.9	184.1	393.4
		0.05	244.2	174.3	389.3
	0.98	All alleles	356.4	286.2	467.4
		No singletons*	292.8	215	445.1
		0.01	292.8	215	445.1
		0.02	254.6	184.3	395.5
		0.05	246.5	176	392.8
91 (without neonates <30cm)	0.7	All alleles	329.2	261.6	439
		No singletons*	294.3	214.1	456.6
		0.01	456.6	214.1	456.6
		0.02	244.7	175.3	388.9
		0.05	235.3	168.7	373.1
	0.9	All alleles	333.9	266.5	442
		No singletons*	291.4	211.9	452.3
		0.01	291.4	211.9	452.3
		0.02	248.2	177.1	397.6
		0.05	244.1	175.2	386
	0.95	All alleles	339.1	270	450.8
		No singletons*	289.7	209.7	453.3
		0.01	289.7	209.7	453.3
		0.02	248.7	177.1	400
		0.05	244.5	174.5	391
	0.98	All alleles	339	269.3	452.3
		No singletons*	288.7	208.7	452.9

		0.01	288.7	208.7	452.9
		0.02	250.5	178.4	402.6
		0.05	248.6	177.9	395.7

Table S6. Estimates of the effective size (*Ne*) and 95% confidence interval (CI) for the angelshark (*S. squatina*) population in Corsica (call rate across loci = 0.95 and without singletons alleles), using the Linkage Disequilibrium method, for varying sample sizes with different age structures.

Sample size	Number of SNPs	*Ne*	JackKnife 95% CI	Parametric 95% CI
100 (all individuals)	9,729	296.6	218.3 - 448.9	294.7 - 298.5
91 (without newborn individuals <30cm)	9,389	289.7	209.7 - 453.3	287.7 - 291.8
78 (only individuals >80cm)	8,838	273.6	190.8 - 462.8	271.3 - 275.8
51 (only mature adults >100cm)	7,741	329.5	189 - 1102.8	324.2 - 335

Table S7. Estimates of the effective size (*Ne*) and 95% confidence interval (CI) for the angelshark (*S. squatina*) population in Corsica (9,389 SNPs across 91 individuals; call rate across loci = 0.95 and without singletons alleles), using the Linkage Disequilibrium method, for each year of sampling.

Sampling year	Number of individuals	Number of SNPs	*Ne*	JackKnife 95% CI	Parametric 95% CI
2021	61	7,980	230.6	156 - 418.9	228.4 - 232.8
2022	30	6,705	176.7	84 - infinite	173.3 - 180.3

Supporting figures

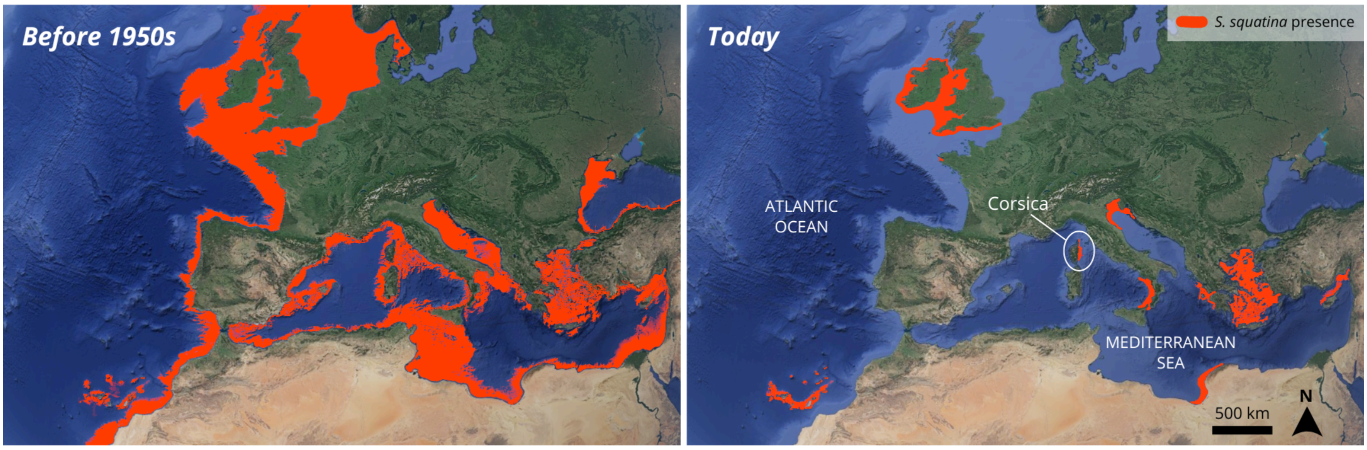

Figure S1. Former and current distribution of the angelshark (*Squatina squatina*) adapted from the IUCN Red List of Threatened Species (Morey *et al*., 2019; Lawson *et al*. 2020). The area of study, Corsica island, is indicated on the right map.

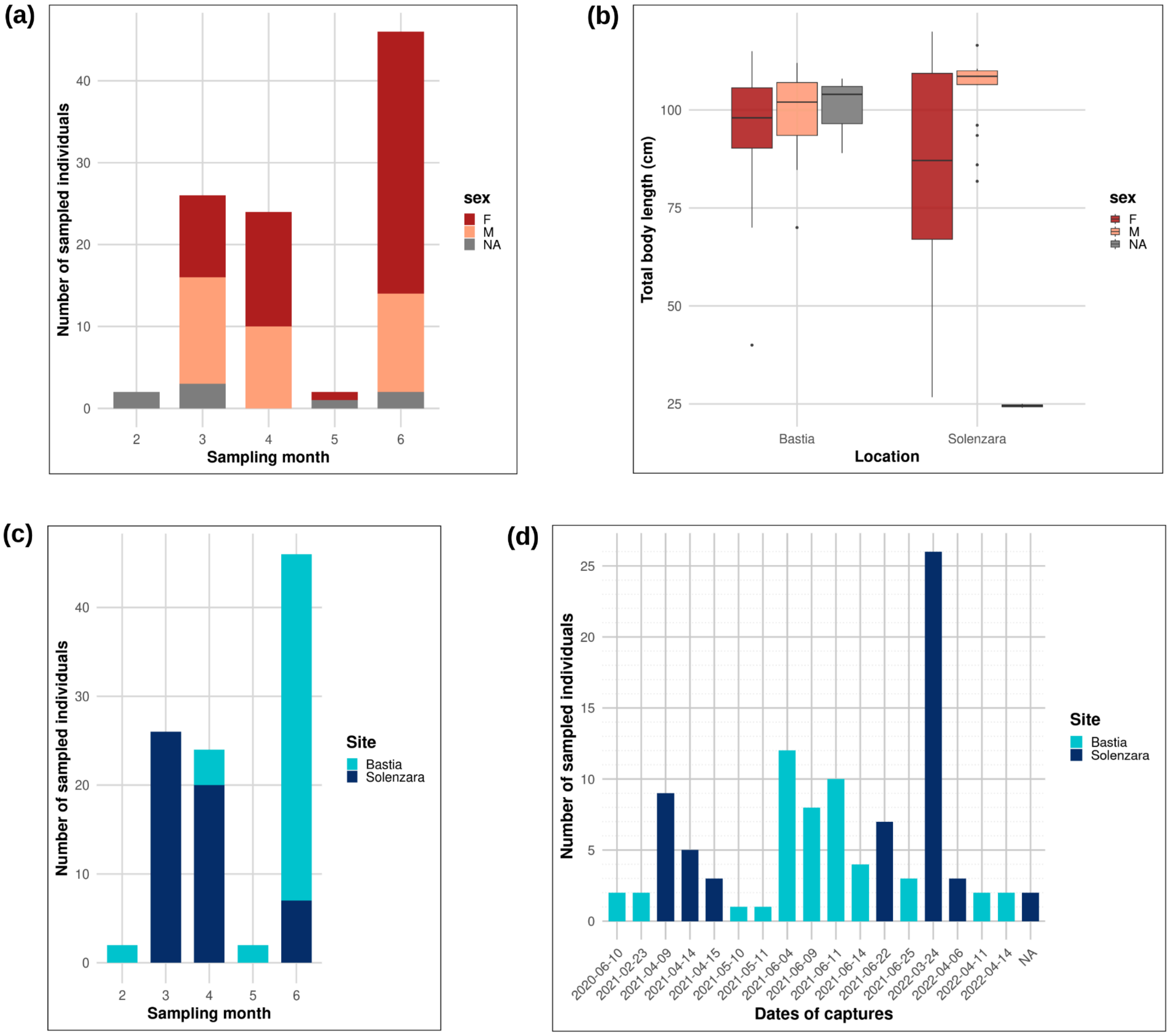

Figure S2. (a) Sex of sampled angelsharks (*Squatina squatina*) individuals caught by fishers depending on the sampling month. (b) Size distribution (total body length, in centimeters) for individuals sampled at two sites (Bastia and Solenzara) on the east coast of Corsica. Samples from Bastia include 27 females, 15 males and 5 unknown, while those from Solenzara include 31 females, 21 males and 6 unknown. (c) Origin of sampled individuals caught by fishers depending on the sampling month. (d) Number of sampled individuals per sampling event

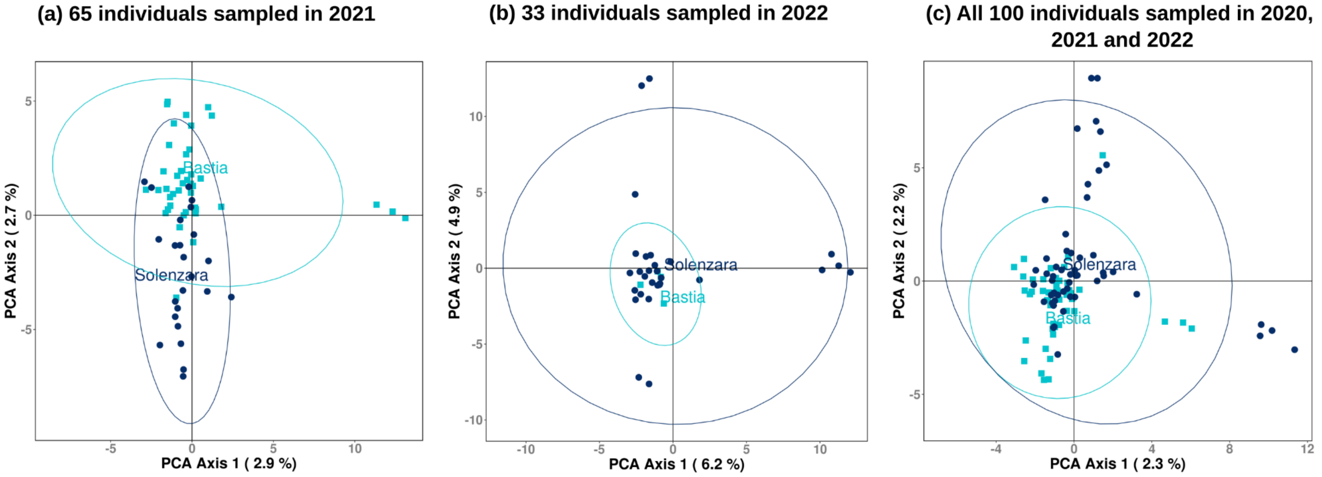

Figure S3. Principal Component Analysis (PCA) with individuals sampled in 2021 (a), sampled in 2022 (b) and sampled across all years (c), in Bastia and Solenzara. The size of the ellipses encompasses 95% of individuals, assuming a *t*-distribution.

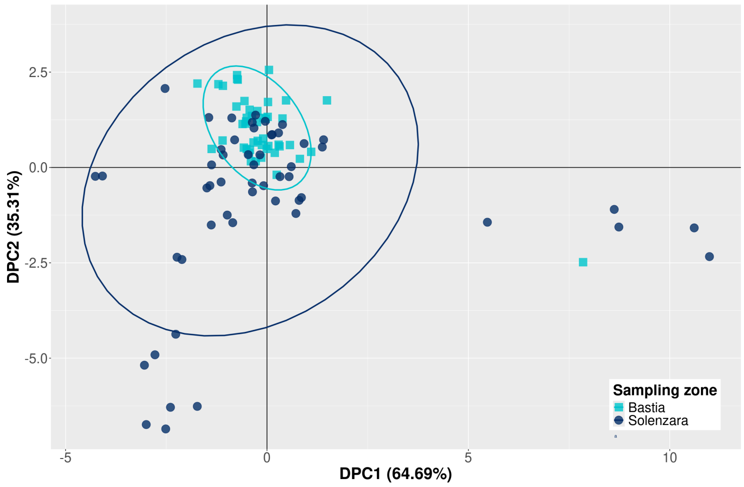

Figure S4. Discriminant Analysis of Principal Components (DAPC) plot of 100 *S. squatina* individuals (each point indicates one individual) sampled in Corsica (Bastia and Solenzara) and genotyped at 9,699 SNPs. Five PCs and two discriminant functions were retained to describe the relationships between clusters. The size of the ellipses encompasses 95% of individuals, assuming a *t*-distribution

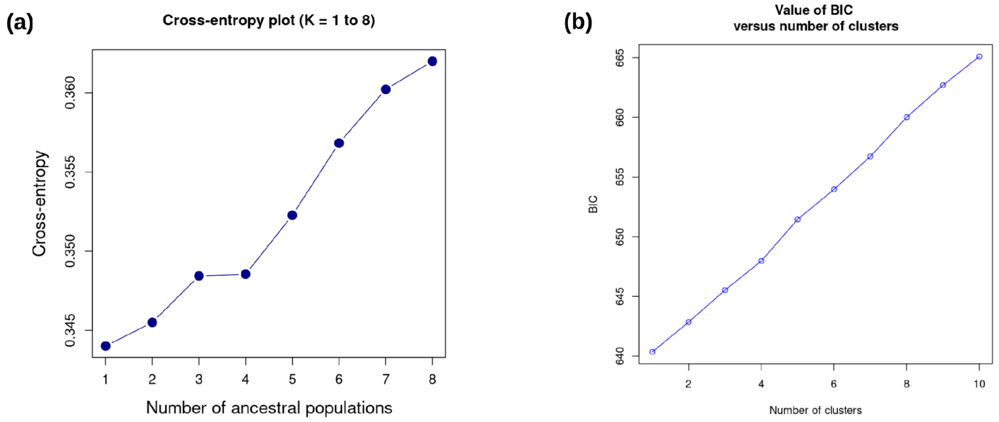

Figure S5. Cross-entropy criterion (a) and Bayesian Information Criterion (BIC, b) for selecting the optimal number of clusters for the method based on sparse non-negative matrix factorization (sNMF) and Discriminant Analysis of Principal Components (DAPC) respectively, for a dataset of 9,699 SNPs across 100 individuals.

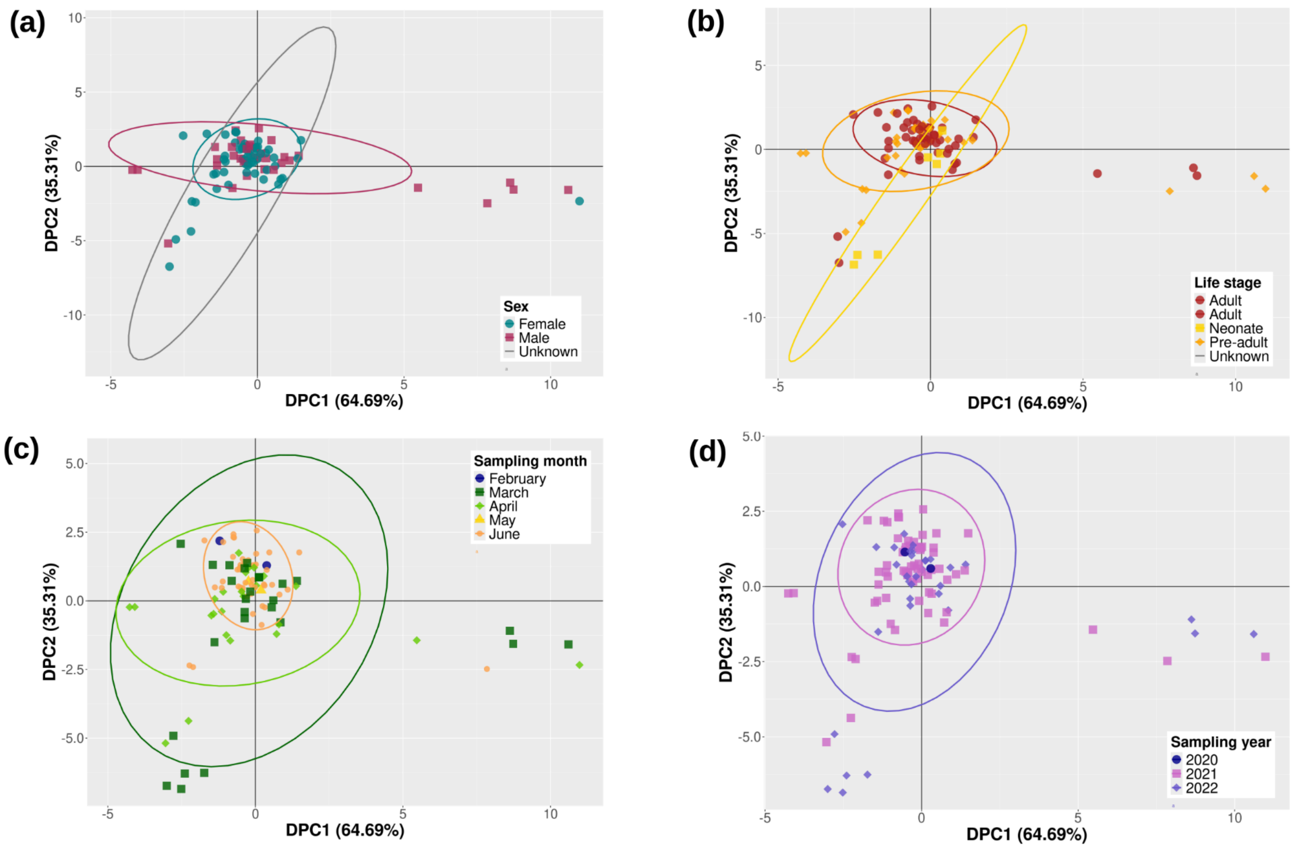

Figure S6. Discriminant Analysis of Principal Components (DAPC) results for the 100 *S. squatina* individuals sampled in Corsica (Bastia and Solenzara) and genotyped at 9,699 SNPs, plotted against the sex (a), life stage (b), sampling month (c) and sampling year (d). The size of the ellipses encompasses 95% of individuals, assuming a *t*-distribution.

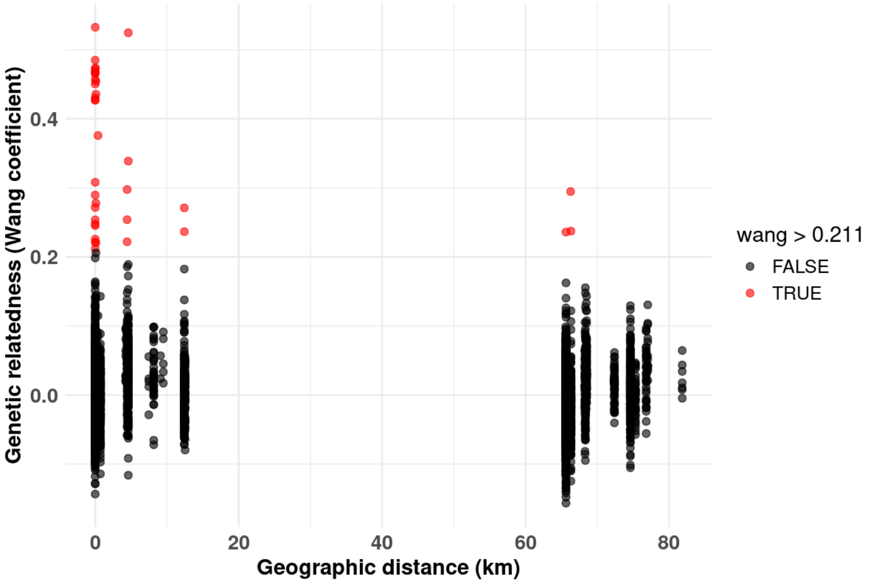

Figure S7. Influence of geographical distance (Euclidean distance) on genetic relatedness (Wang coefficient) between all pairs of angelshark individuals. Pairs of individuals with a Wang genetic relatedness coefficient ≥0.211 (the lower bond of the confidence interval estimated for half-siblings) are coloured red.

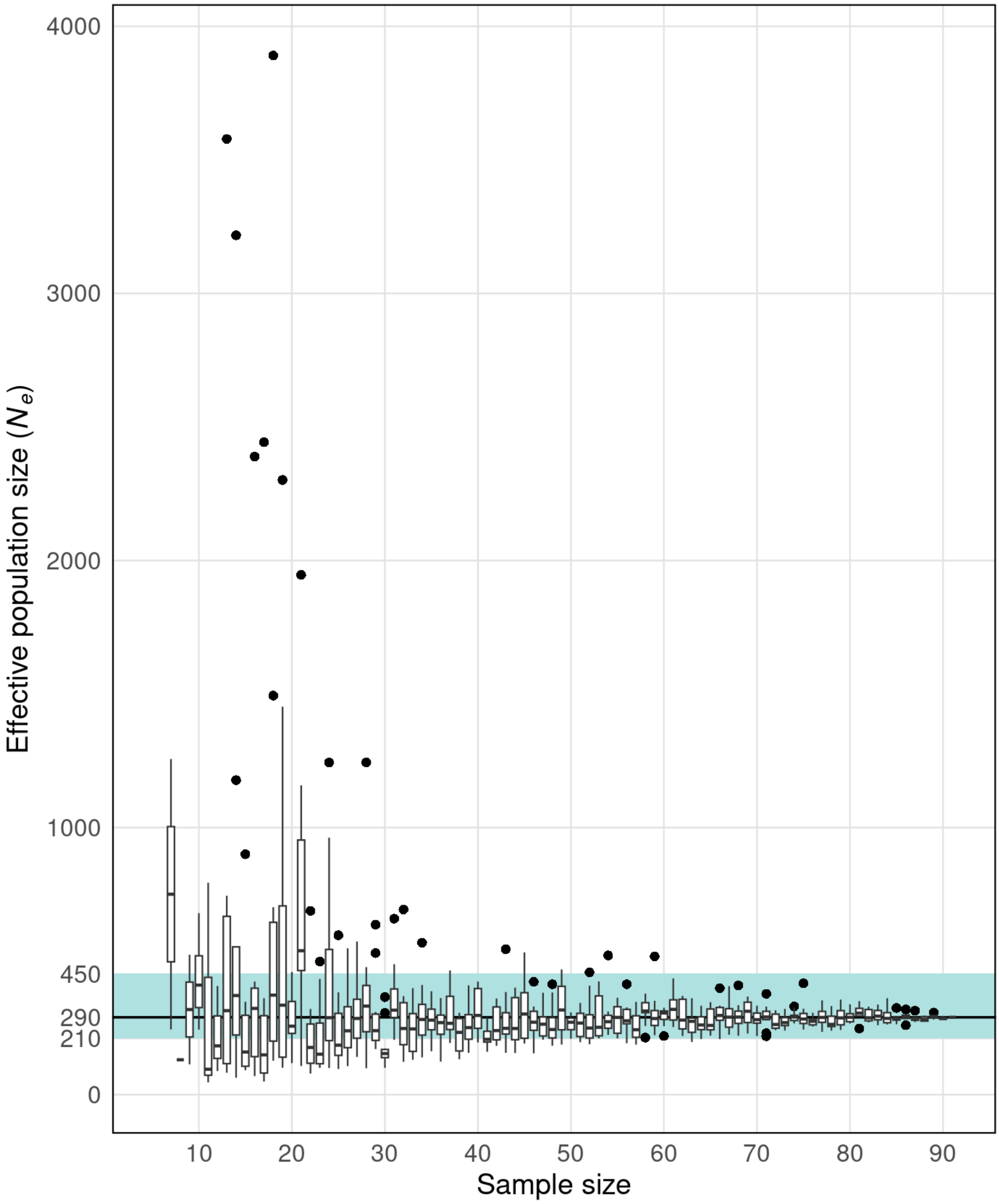

Figure S8. Influence of sample size (number of individuals sampled) on the precision of the effective population size (*Ne*) estimate. For each sample size (5 to 91), the *Ne* was calculated 10 times using randomly selected individuals. The black line represents the *Ne* value of 290, calculated with 91 individuals. The blue shaded area (210–450) shows the 95% confidence interval for *Ne*=290, estimated using the Jackknife method for the sample size of 91 individuals.

References

Awruch, C. A., Nostro, F. L., Somoza, G. M., & Di Giácomo, E. (2008). Reproductive biology of the angular angel shark *Squatina guggenheim* (Chondrichthyes: Squatinidae) off Patagonia (Argentina, southwestern Atlantic). *Ciencias Marinas*, *34*(1), 17-28. http://dx.doi.org/10.7773/cm.v34i1.1232

Baremore, I. E. (2010). Reproductive aspects of the Atlantic angel shark *Squatina dumeril*. *Journal of Fish Biology*, *76*(7), 1682-1695. https://doi.org/10.1111/j.1095-8649.2010.02608.x

Bousquet, C., Mourier, J., Giovos, I., Meyers, E. K., Dijoux, J., & Durieux, E. D. (2024). Local Ecological Knowledge and Fishery Data Provides Important Information on the Distribution and Seasonal Dynamic of Critically Endangered Angel Sharks in Corsica (Mediterranean Sea, France). *Available at SSRN 4872813*. https://dx.doi.org/10.2139/ssrn.4872813

Cailliet, G. M., Mollet, H. F., Pittenger, G. G., Bedford, D., & Natanson, L. J. (1992). Growth and demography of the Pacific angle shark (*Squatina californica*), based upon tag returns off California. *Marine and Freshwater Research*, *43*(5), 1313-1330. http://dx.doi.org/10.1071/MF9921313

Capapé, C., Quignard, J. P., & Mellinger, J. (1990). Reproduction and development of two angel sharks, *Squatina squatina* and *S. oculata* (Pisces: Squatinidae), off Tunisian coasts: Semi-delayed vitellogenesis, lack of egg capsules, and lecithotrophy. *Journal of Fish Biology*, *37*(3), 347-356. https://doi.org/10.1111/j.1095-8649.1990.tb05865.x

Cavallaro, M., Ammendolia, G., & Navarra, E. (2015). Finding of a rare *Squatina squatina* (Linnaeus, 1758) (Chondrichthyes: Squatinidae) along the Tyrrhenian coast of the Strait of Messina and its maintenance in an aquarium. *Marine Biodiversity Records*, *8*, e44. http://dx.doi.org/10.1017/S1755267215000226

Compagno, L. J. V. (1984). FAO species catalogue. Sharks of the world: an annotated and illustrated catalogue of shark species known to date. Part 1: Hexanchiformes to Lamniformes. FAO Fisheries Synopsis No. 125, 4 (1):1–250.

Ellis, J. R., Barker, J., McCully Phillips, S. R., Meyers, E. K., & Heupel, M. (2021). Angel sharks (Squatinidae): A review of biological knowledge and exploitation. *Journal of Fish Biology*, *98*(3), 592-621. https://doi.org/10.1111/jfb.14613

Frichot, E., & François, O. (2015). LEA: An R package for landscape and ecological association studies. *Methods in Ecology and Evolution*, *6*(8), 925-929. https://doi.org/10.1111/2041-210X.12382

Lawson, J. M., Pollom, R. A., Gordon, C. A., Barker, J., Meyers, E. K., Zidowitz, H., ... & Dulvy, N. K. (2020). Extinction risk and conservation of critically endangered angel sharks in the Eastern Atlantic and Mediterranean Sea. *ICES Journal of Marine Science*, *77*(1), 12-29. http://dx.doi.org/10.1093/icesjms/fsz222

Lo Bianco, S. (1899). Notizie biologiche riguardanti specialmente il periodo di maturità sessuale degli animali del golfo di Napoli. Mittheilungen aus der Zoologischen Station zu Neapel, 13, 448–573.

Morey, G., Barker, J., Hood, A., Gordon, C., Bartolí, A., Meyers, E. K. M., ... & Pollom, R. (2019). *Squatina squatina*. *The IUCN Red List of Threatened Species*, *2019*, e-T39332A11749837.

Osaer, F., Narváez, K., Pajuelo, J. G., & Lorenzo, J. M. (2015). Sexual development and maturity scale for the angel shark *Squatina squatina* (Elasmobranchii: Squatinidae), with comments on the adequacy of general maturity scales. *Sexuality and Early Development in Aquatic Organisms*, 1, 117–132. http://dx.doi.org/10.3354/sedao00012
